# Causal relationship between the plasma lipidome and urological cancers: A two-sample Mendelian randomization study

**DOI:** 10.1097/MD.0000000000042577

**Published:** 2025-06-06

**Authors:** Rong Chen, Liujie He, Zhiyao Huang, Jie Sun, Qiang Wang

**Affiliations:** a Department of Graduate, Hebei North University, Zhangjiakou, Hebei Province, China; b Naval Medical University, Shanghai, China; c Department of Urology, Peking University People’s Hospital, Beijing, China.

**Keywords:** bladder cancer, kidney cancer, Mendelian randomization, plasma lipidome, prostate cancer

## Abstract

The plasma lipidome has been found to be closely related to inflammation, obesity, aging, and other diseases, including various cancers. However, the causal relationship between specific plasma lipid species and the risk of urological cancers remains unclear. This study aimed to investigate the causal relationship between 179 lipid species and 3 common types of urological cancers using a two-sample Mendelian randomization (MR) approach. Large-scale genome-wide association study datasets of 179 lipid species were analyzed to evaluate the association between the plasma lipidome and urological cancers, including bladder cancer, kidney cancer, and prostate cancer. The inverse variance weighted method was employed as the primary analysis approach, supplemented by MR-Egger regression, weighted median, weighted mode, and simple mode methods to ensure the reliability of the findings. Sensitivity analyses, including Cochran *Q* test, MR-Egger intercept test, MR Pleiotropy Residual Sum And Outlier, and leave-one-out analysis, were performed to assess the stability and robustness of the causal relationship. The inverse variance weighted analysis showed that higher levels of sterol ester (SE) (27:1/16:0) and other plasma lipidome were causally associated with increased bladder cancer risk. Similarly, elevated levels of phosphatidylcholine (16:0_22:6) and others were linked to increased kidney cancer risk. For prostate cancer, higher levels of SE (27:1/17:0) and others were associated with increased risk, while higher levels of SE (27:1/18:2) and others were associated with decreased risk. The study provides strong evidence for the causal relationships between plasma lipidome and bladder, kidney, and prostate cancers. These findings illuminate complex lipid metabolism pathways in urinary system cancer etiology and highlight specific lipid structures’ differential impact on cancer risk. This research lays a foundation for further exploring biological mechanisms and developing early screening tools and targeted therapies for urological cancers.

## 1. Introduction

Bladder cancer, kidney cancer, and prostate cancer are the 3 most prevalent malignancies of the urinary system, imposing a significant global health burden. Bladder cancer ranks as the 10th most common cancer worldwide,^[1]^ with 613,791 new cases and 220,349 deaths reported in 2022.^[2]^ Kidney cancer, recognized as one of the most malignant tumors in the urinary system, has shown increasing morbidity and mortality globally, accounting for 434,419 new cases and 155,702 deaths in 2022.^[2,3]^ Prostate cancer stands as the second most frequently diagnosed cancer and the fifth leading cause of cancer death among men, with 1466,680 new cases and 396,792 deaths in 2022.^[2,4]^ While established risk factors such as smoking, carcinogen exposure, and genetic predisposition are well-documented for urological cancers,^[1–4]^ emerging evidence highlights the critical role of lipid metabolism in the onset and progression of these malignancies.^[5,6]^ In bladder cancer, upregulation of sterol regulatory element-binding protein 1 and fatty acid synthase enhance lipid synthesis, thereby promoting tumor proliferation, migration, and treatment resistance.^[7]^ Kidney cancer, particularly clear cell renal cell carcinoma, is characterized by significant lipid accumulation. Scavenger receptor B1 facilitates cholesterol transport into cancer cells via high-density lipoprotein, thereby enhancing cell viability and tumor growth.^[8]^ Furthermore, increased expression of CDK13 promotes fatty acid synthesis and lipid deposition, advancing prostate cancer progression.^[9]^ These findings indicate that targeting lipid metabolism pathways could be a potential therapeutic strategy for urological cancers.

Lipids are a class of hydrophobic or amphipathic molecules involved in energy metabolism, signal transduction, and cellular structure maintenance, making them essential for life.^[10,11]^ The complete lipid composition of a cell, known as the lipidome, is vast, with the number of lipid species estimated to range from tens of thousands to even millions, present at levels from amol/mg to nmol/mg protein.^[12]^ Although lipidomics is a relatively recent field, advancements in mass spectrometry have significantly enhanced its application in biomedical research.^[10,12,13]^ Increasing evidence links the plasma lipidome to the development and progression of various cancers.^[5,14,15]^ Several studies suggest that plasma lipid profiles may serve as early detection biomarkers for multiple cancer types.^[14,16,17]^ Therefore, we believe that it is necessary to explore the causal relationship between plasma lipidome and urological cancers.

Mendelian randomization (MR) uses genetic variants strongly associated with exposure factors as instrumental variables (IVs) to assess the causal relationship between exposure factors and outcomes, thereby reducing confounding factors in traditional observational studies.^[18,19]^ Additionally, since genetic variants are present from birth, the associations identified through MR are not influenced by reverse causation.^[20,21]^ In this study, we employed MR analysis to explore the causal relationship between 179 lipid species and urinary system cancers, aiming to inform treatment research and early screening for common urological cancers.

## 2. Materials and methods

### 2.1. Study design

The two-sample MR approach was used to analyze the causal relationship between plasma lipidome and urinary system cancer, with plasma lipidome as the exposure factor and urinary system cancer as the outcome. Sensitivity analyses were conducted to evaluate the stability and reliability of this relationship. This MR analysis method is based on 3 key assumptions: (1) genetic variations are strongly associated with exposure, (2) genetic variations are not associated with other confounders, and (3) genetic variations are not directly associated with outcomes.^[18,19]^ The study was a secondary analysis of published human data and did not require ethical approval.

### 2.2. Data sources for exposure and outcome data

#### 2.2.1. Exposure data

The plasma lipidome data used in this study were obtained from a pooled genome-wide association study (GWAS) analysis of 179 lipid species (GCST90277238–GCST90277416) involving 7174 Finnish individuals.^[13]^ These GWAS summary statistics provided the genetic instruments for the plasma lipidome exposures in the MR analyses.

#### 2.2.2. Outcome data

The outcome data for this study were derived from the FinnGen database, focusing on 3 urinary system cancers: bladder cancer, prostate cancer, and kidney cancer. For bladder cancer, the dataset included 2193 cases and 314,193 controls (finngen_R10_C3_BLADDER_EXALLC). The kidney cancer dataset consisted of 2372 cases and 314,193 controls (finngen_R10_C3_KIDNEY_NOTRENALPELVIS_EXALLC). Lastly, the prostate cancer dataset included 15,199 cases and 131,266 controls (finngen_R10_C3_PROSTATE_EXALLC). Utilizing GWAS summary statistics from the Finnish population ensures a consistent genetic background for the MR analyses, thereby minimizing the potential for population stratification bias.

### 2.3. Selection of IVs

To identify single nucleotide polymorphisms (SNPs) strongly associated with the plasma lipidome exposures, we applied a significance threshold of *P* < 1 × 10⁻⁵ using the pooled GWAS summary statistics for the plasma lipidome and urinary system cancers. One of the key principles of the MR method is to ensure no linkage disequilibrium between the selected SNPs. Therefore, we set a distance of 10,000 kb and a linkage disequilibrium threshold of *r*² < 0.001 to minimize bias from residual linkage disequilibrium of genetic variation. Furthermore, we extracted the beta coefficients and standard errors for each SNP to calculate the F-statistic. SNPs with an F-statistic > 10 were retained to avoid errors arising from weak instrument bias.

### 2.4. Statistical analysis

We utilized the inverse variance weighting (IVW) method as our primary analysis approach, supplemented by MR-Egger regression, weighted median, weighted mode, and simple modal methods.^[22,23]^ A causal relationship was considered statistically significant if the IVW method yielded a *P*-value <.05 and the direction of the beta coefficient was consistent across all 5 MR methods.^[24]^ Odds ratios (ORs) and their corresponding 95% confidence intervals (CIs) were calculated to determine whether the exposure was a risk factor or a protective factor for the outcome. Comprehensive sensitivity analyses were performed to verify the stability of the results. Horizontal pleiotropy was assessed using the MR-Egger regression test; horizontal pleiotropy was indicated if the intercept term was significant (*P* ≤ .05).^[25,26]^ The MR-Egger regression method incorporates an intercept term in its model and weights the analysis using the inverse of the squared standard errors of the genetic associations with the outcome. The Cochran *Q* test was used to determine the heterogeneity of SNPs.^[22]^ The Cochran *Q* test is a non-parametric statistical method used to compare the proportions of a binary variable across 3 or more related samples. Significant heterogeneity was indicated by a Cochran *Q* test *P*-value below the predetermined alpha of .05. Funnel plots were employed to visualize potential heterogeneity in the results. Leave-one-out analysis was used to assess whether significant results were determined by a single SNP. The MR Pleiotropy Residual Sum and Outlier (MR-PRESSO) method was employed to identify outliers contributing to horizontal pleiotropy in multi-instrument summary-level MR analyses.^[27]^ The MR-PRESSO global test assesses the overall horizontal pleiotropy across all IVs in a MR analysis by comparing the observed sum of squared residuals of genetic variants relative to the regression line against their expected distribution under the null hypothesis of no horizontal pleiotropy. This approach detects and removes pleiotropic IVs that may bias the causal effect estimates in MR studies.^[24]^ By iteratively identifying and removing outliers, MR-PRESSO reevaluates the causal effects after accounting for pleiotropy, providing a more robust assessment of the relationship between the exposure and the outcome.^[24]^ Additionally, we excluded genetic variants that demonstrated significant associations with established confounding factors related to lipid metabolism and cancer risk. All MR analyses were performed with the use of R software (version 4.3.3) with the “TwoSampleMR” package, and the “ggplot2” package was used for graphical presentations.

## 3. Results

### 3.1. Causal relationship between plasma lipidome and bladder cancer

**Figure 1.**
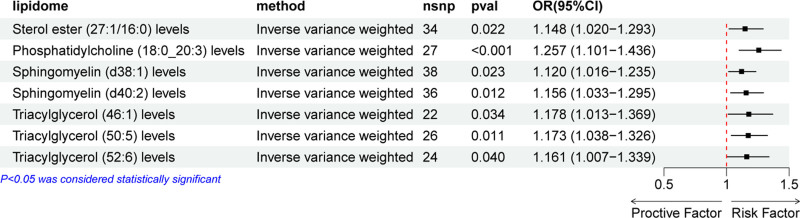
Forest plot displaying the effect of each significant lipid species on bladder cancer. An OR value >1 is considered a risk factor for bladder cancer, while an OR value <1 is considered a protective factor. ORs = odds ratios.

**Figure 2.**
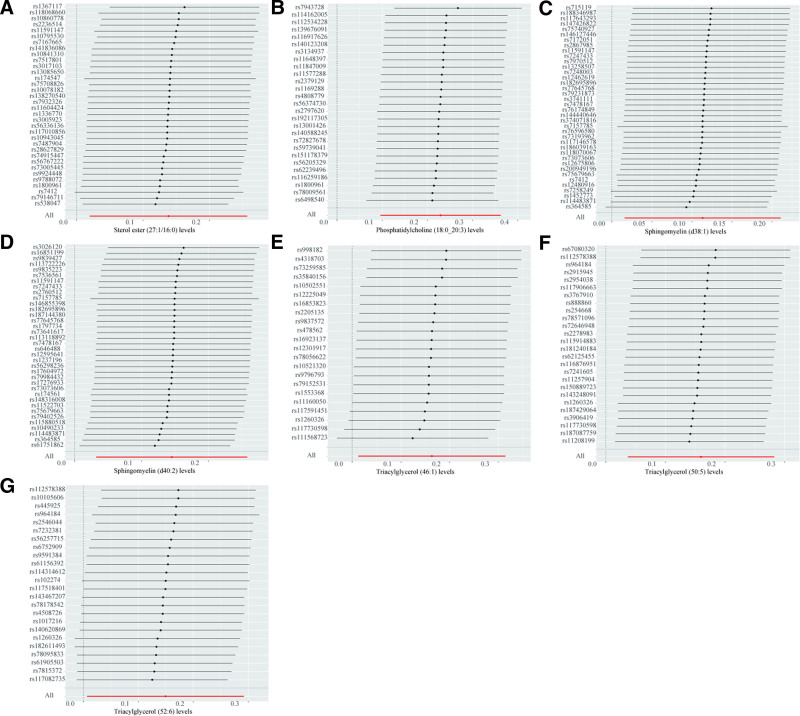
Leave-one-out sensitivity analysis was performed on the lipids found to have significant effects on bladder cancer in the MR analysis. (A) Sterol ester (27:1/16:0) levels, (B) phosphatidylcholine (18:0_20:3) levels, (C) sphingomyelin (d38:1) levels, (D) sphingomyelin (d40:2) levels, (E) triacylglycerol (46:1) levels, (F) triacylglycerol (50:5) levels, (G) triacylglycerol (52:6) levels. MR = Mendelian randomization.

A two-sample MR analysis was performed to investigate the causal relationship between the plasma lipidome and bladder cancer. As depicted in Figure 1, several lipid species were significantly associated with an increased risk of bladder cancer. Specifically, higher levels of sterol ester (SE) (27:1/16:0) (OR = 1.148, 95% CI = 1.020–1.293, *P* = .022), phosphatidylcholine (PC) (18:0_20:3) (OR = 1.257, 95% CI = 1.101–1.436, *P* < .001), sphingomyelin (SM) (d38:1) (OR = 1.120, 95% CI = 1.016–1.235, *P* = .023), SM (d40:2) (OR = 1.156, 95% CI = 1.033–1295, *P* = .012), triacylglycerol (TG) (46:1) (OR = 1.178, 95% CI = 1.013–1.369, *P* = .034), TG (50:5) (OR = 1.173, 95% CI = 1.038–1.326, *P* = .011), and TG (52:6) (OR = 1.161, 95% CI = 1.007–1.339, *P* = .040) were causally associated with an increased risk of bladder cancer. However, no lipid species were identified in our analysis that exhibited a protective effect against bladder cancer. The forest plot in Figure 1 displays the results of the IVW method exclusively, while the outcomes of the other 4 methods are detailed in Figure S1, Supplemental Digital Content, https://links.lww.com/MD/P82. The results of the leave-one-out method are detailed in Figure 2.

### 3.2. Causal relationship between plasma lipidome and kidney cancer

**Figure 3.**
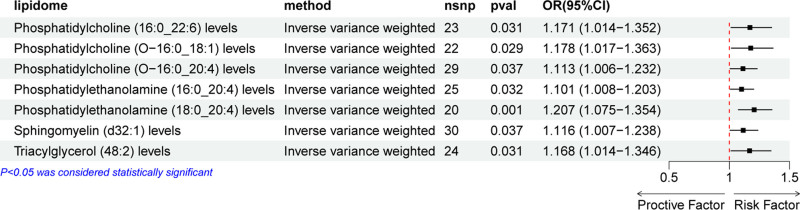
Forest plot displaying the effect of each significant lipid species on kidney cancer. OR values >1 indicate risk factors for kidney cancer, while OR values <1 suggest protective factors. ORs = odds ratios.

We conducted a two-sample MR analysis to explore the causal relationship between the plasma lipidome and kidney cancer. As illustrated in Figure 3, the results identified several lipid species that were significantly associated with an increased risk of kidney cancer. Specifically, higher levels of PC (16:0_22:6) (OR = 1.171, 95% CI = 1.014–1.352, *P* = .031), PC (O-16:1_18:1) (OR = 1.178, 95% CI = 1.017–1.363, *P* = .029), PC (O-16:0_20:4) (OR = 1.113, 95% CI = 1.006–1.232, *P* = .037), phosphatidylethanolamine (PE) (16:0_20:4) (OR = 1.101, 95% CI = 1.008–1.203, *P* = .032), PE (18:0_20:4) (OR = 1.207, 95% CI = 1.075–1.354, *P* = .001), SMs (d32:1) (OR = 1.116, 95% CI = 1.007–1.238, *P* = .037), and TG (48:2) (OR = 1.168, 95% CI = 1.014–1.346, *P* = .031) were causally associated with an increased risk of kidney cancer.

**Figure 4.**
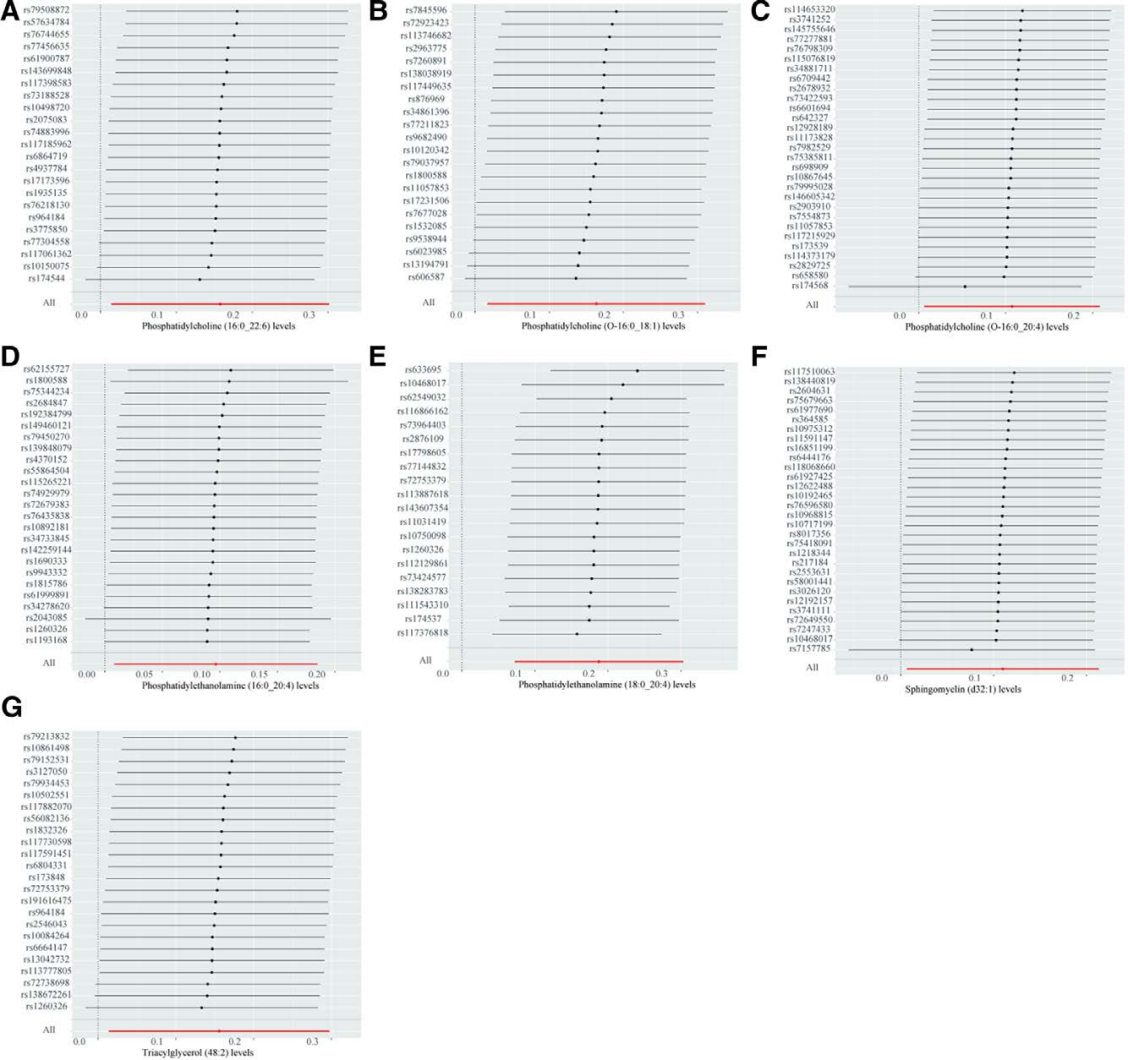
Leave-one-out sensitivity analysis was performed on the lipids found to have significant effects on kidney cancer in the MR analysis. (A) Phosphatidylcholine (16:0_22:6) levels, (B) phosphatidylcholine (O-16:1_18:1) levels, (C) phosphatidylcholine (O-16:0_20:4) levels, (D) phosphatidylethanolamine (16:0_20:4), (E) phosphatidylethanolamine (18:0_20:4) levels, (F) sphingomyelin (d32:1) levels, (G) triacylglycerol (48:2) levels. MR = Mendelian randomization.

Similar to the findings in bladder cancer, no lipid species were identified that exhibited a protective effect against kidney cancer. In the forest plot (Fig. 3), only the results of the IVW method are shown, and the results of the other 4 methods can be viewed in Figure S4, Supplemental Digital Content, https://links.lww.com/MD/P82. The results obtained from the leave-one-out approach are shown in Figure 4.

### 3.3. Causal relationship between plasma lipidome and prostate cancer

**Figure 5.**
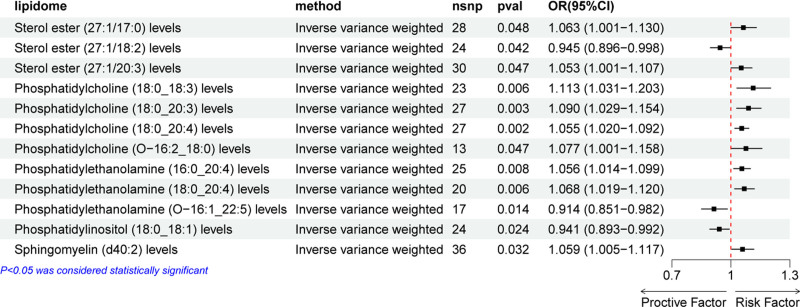
Forest plot displaying the effect of each significant lipid species on prostate cancer. An OR value >1 is considered a risk factor for prostate cancer, while an OR value <1 is considered a protective factor. ORs = odds ratios.

A two-sample MR analysis was conducted to explore the causal relationship between the plasma lipidome and prostate cancer. As presented in Figure 5, the results identified several lipid species significantly associated with an increased risk of prostate cancer. Specifically, higher levels of SE (27:1/17:0) (OR = 1.063, 95% CI = 1.001–1.130, *P* = .048), SE (27:1/20:3) (OR = 1.053, 95% CI = 1.000–1.107, *P* = .047), PC (18:0_18:3) (OR = 1.113, 95% CI = 1.031–1.203, *P* = .006), PC (18:0_20:3) (OR = 1.090, 95% CI = 1.029–1.154, *P* = .003), PC (18:0_20:4) (OR = 1.055, 95% CI = 1.020–1.092, *P* = .002), PC (O-16:2_18:0) (OR = 1.077, 95% CI = 1.001–1.158, *P* = .047), PE (16:0_20:4) (OR = 1.056, 95% CI = 1.014–1.099, *P* = .008), PE (18:0_20:4) (OR = 1.068, 95% CI = 1.019–1.120, *P* = .006), and SM (d40:2) (OR = 1.059, 95% CI = 1.005–1.117, *P* = .032) were causally associated with an increased risk of prostate cancer.

**Figure 6.**
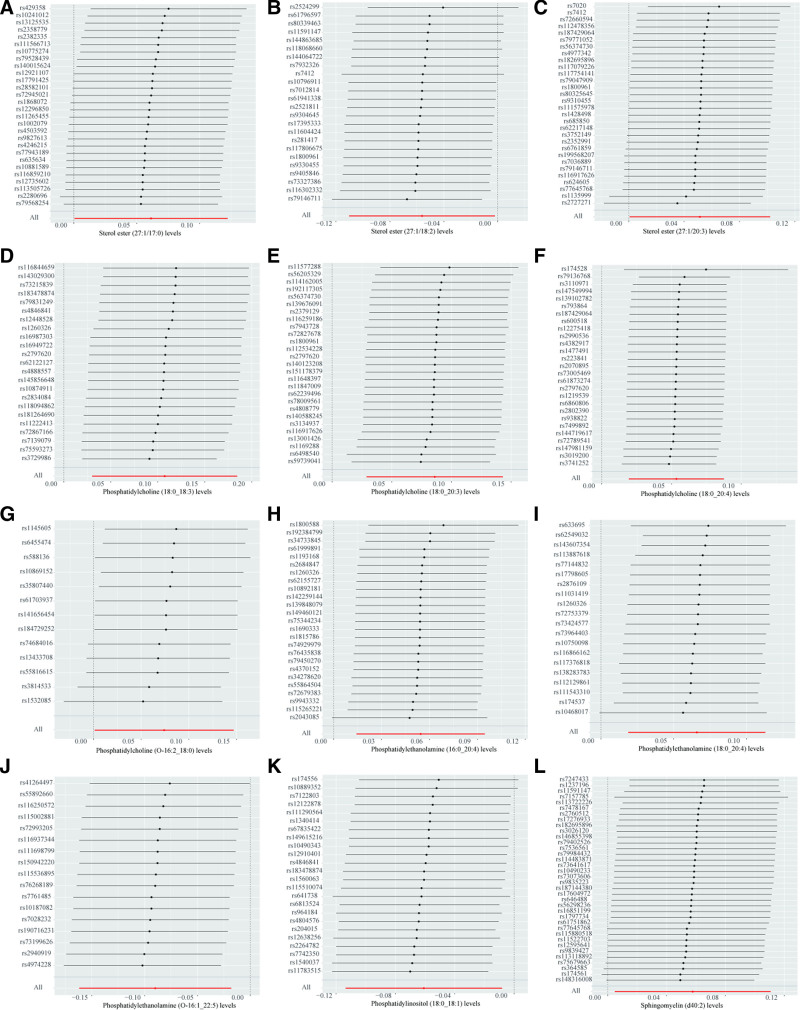
Leave-one-out sensitivity analysis was performed on the lipids found to have significant effects on prostate cancer in the MR analysis. (A) Sterol ester (27:1/17:0) levels, (B) sterol ester (27:1/18:2) levels, (C) sterol ester (27:1/20:3) levels, (D) phosphatidylcholine (18:0_18:3) levels, (E) phosphatidylcholine (18:0_20:3) levels, (F) phosphatidylcholine (18:0_20:4) levels, (G) phosphatidylcholine (O-16:2_18:0) levels, (H) phosphatidylethanolamine (16:0_20:4) levels, (I) phosphatidylethanolamine (18:0_20:4), (J) phosphatidylethanolamine (O-16:1_22:5) levels, (K) phosphatidylinositol (18:0_18:1) levels, (L) sphingomyelin (d40:2) levels. MR = Mendelian randomization.

In contrast, the analysis also revealed that certain lipid species exhibited a protective effect against prostate cancer. Higher levels of SE (27:1/18:2) (OR = 0.945, 95% CI = 0.896–0.998, *P* = .042), PE (O-16:1_22:5) (OR = 0.914, 95% CI = 0.851–0.982, *P* = .014), and phosphatidylinositol (PI) (18:0_18:1) (OR = 0.941, 95% CI = 0.893–0.992, *P* = .024) were associated with a decreased risk of prostate cancer. The forest plot in Figure 5 displays the results of the IVW method exclusively, while the outcomes of the other 4 methods can be viewed in Figure S7, Supplemental Digital Content, https://links.lww.com/MD/P82. Figure 6 displays the outcomes derived using the leave-one-out method.

### 3.4. Sensitivity analyses

Comprehensive sensitivity analyses were conducted to evaluate the robustness of the findings. Heterogeneity was found in the causal relationship between PC (20:4_0:0) levels (Cochran Q_pval = 0.039) and PE (O-16:1_18:2) levels (Cochran Q_pval = 0.049) with prostate cancer. Consequently, we excluded the association between these 2 lipids and prostate cancer from further analysis. For all other identified causal relationships, no evidence of heterogeneity was found (Table 1). To assess the presence of horizontal pleiotropy, the MR-Egger regression test was employed (Table 1). A significant intercept term in the MR-Egger analysis (*P* < .05) would indicate the presence of horizontal pleiotropy. However, all *P*-values derived from the MR-Egger regression test were >.05, suggesting the absence of pleiotropy in our analyses. Furthermore, the MR-PRESSO test did not detect any instances of pleiotropy, further confirming the validity of our findings. Scatter plots of the MR results are presented in Figures S2, S5, and S8, Supplemental Digital Content, https://links.lww.com/MD/P82. The funnel plots all show no significant heterogeneity (Figures S3, S6, and S9, Supplemental Digital Content, https://links.lww.com/MD/P82). Leave-one-out analysis was used to assess whether significant results were determined by a single SNP (Figs. 2, 4, and 6). Although some individual SNPs were found to influence the results, the odds ratio consistently remained on the same side of the zero line, indicating that no single SNP drove the observed associations.

**Table 1:** Sensitivity test results.

Outcome	Exposure	Cochran *Q*			Pleiotropy		
		Q	Q_df	Q_pval	Egger_intercept	SE	*P*-val
Bladder cancer	Sterol ester (27:1/16:0) levels	39.833	33.000	0.192	-0.014	0.016	.396
	Phosphatidylcholine (18:0_20:3) levels	15.930	26.000	0.938	0.004	0.023	.867
	Sphingomyelin (d38:1) levels	31.765	37.000	0.713	-0.013	0.015	.388
	Sphingomyelin (d40:2) levels	36.054	35.000	0.419	-0.019	0.016	.249
	Triacylglycerol (46:1) levels	18.600	21.000	0.611	-0.036	0.023	.141
	Triacylglycerol (50:5) levels	21.796	25.000	0.648	-0.001	0.019	.970
	Triacylglycerol (52:6) levels	25.563	23.000	0.322	-0.019	0.023	.405
Kidney cancer	Phosphatidylcholine (16:0_22:6) levels	10.847	22.000	0.977	0.002	0.020	.920
	Phosphatidylcholine (O-16:0_18:1) levels	18.532	21.000	0.615	0.003	0.022	.910
	Phosphatidylcholine (O-16:0_20:4) levels	19.160	28.000	0.893	-0.004	0.013	.788
	Phosphatidylethanolamine (16:0_20:4) levels	17.244	24.000	0.838	0.013	0.016	.413
	Phosphatidylethanolamine (18:0_20:4) levels	28.042	19.000	0.083	0.008	0.029	.778
	Sphingomyelin (d32:1) levels	14.522	29.000	0.988	0.014	0.014	.318
	Triacylglycerol (48:2) levels	11.498	23.000	0.978	0.001	0.023	.974
Prostate cancer	Sterol ester (27:1/17:0) levels	27.359	27.000	0.445	0.001	0.010	.883
	Sterol ester (27:1/18:2) levels	16.801	23.000	0.819	0.002	0.008	.759
	Sterol ester (27:1/20:3) levels	26.918	29.000	0.576	-0.003	0.009	.765
	Phosphatidylcholine (20:4_0:0) levels	39.994	26.000	0.039	-0.002	0.008	.816
	Phosphatidylcholine (18:0_18:3) levels	30.714	22.000	0.102	0.014	0.010	.162
	Phosphatidylcholine (18:0_20:3) levels	24.533	26.000	0.546	-0.003	0.010	.777
	Phosphatidylcholine (18:0_20:4) levels	28.758	26.000	0.322	0.004	0.005	.403
	Phosphatidylcholine (O-16:2_18:0) levels	10.277	12.000	0.592	0.003	0.013	.814
	Phosphatidylethanolamine (16:0_20:4) levels	22.091	24.000	0.574	0.002	0.007	.830
	Phosphatidylethanolamine (18:0_20:4) levels	22.312	19.000	0.269	-0.015	0.012	.204
	Phosphatidylethanolamine (O-16:1_18:2) levels	27.674	17.000	0.049	0.015	0.013	.268
	Phosphatidylethanolamine (O-16:1_22:5) levels	8.072	16.000	0.947	0.016	0.012	.199
	Phosphatidylinositol (18:0_18:1) levels	23.774	23.000	0.416	-0.006	0.009	.564
	Sphingomyelin (d40:2) levels	42.831	35.000	0.170	-0.006	0.008	.403

SE = standard error.

## 4. Discussions

Abnormal lipid metabolism is a significant feature of a variety of tumors, and the occurrence and development of tumors are accompanied by changes in plasma lipids, which are of great significance for the early screening and prevention of tumors.^[28–30]^ The use of MR is particularly meaningful in this context, as it allows for the assessment of causal relationships between exposures and outcomes while minimizing the influence of confounding factors and reverse causation.^[31]^ This approach strengthens the validity of the findings and provides a more reliable basis for understanding the complex interplay between lipid metabolism and cancer biology.

This study investigated the causal associations between a wide range of plasma lipid species and the risk of 3 common urinary system cancers: bladder cancer, kidney cancer, and prostate cancer. The findings suggest that specific lipids within the PC, PE, TG, SM, PI, and SE classes may play a role in the development of these malignancies. Seven lipids were found to be associated with bladder cancer, 7 with kidney cancer, and 12 with prostate cancer. Interestingly, we found that PC (18:0_20:3) levels and SM (d40:2) levels were common risk factors for bladder cancer and prostate cancer. Additionally, the levels of PE (18:0_20:4) and PE (16:0_20:4) were common risk factors for both prostate cancer and kidney cancer.

PCs are the most abundant phospholipids in cell membranes and play a crucial role in maintaining membrane integrity and fluidity.^[11]^ Elevated PC metabolism is an important hallmark of cancer, and differences in PC metabolite levels can be detected in the early stages of carcinogenesis.^[32–34]^ Alterations in the levels of specific PC species may affect the biophysical properties of cancer cell membranes, influencing their permeability, receptor signaling, and interactions with the tumor microenvironment.^[35,36]^ PC are metabolized by phospholipase A2, cyclooxygenase-2, lysophosphatidylcholine acyltransferase, and autotaxin to produce prostaglandin E2 (PGE2), platelet-activating factor (PAF), and lysophosphatidic acid (LPA).^[35]^ These lipid mediators bind to their respective receptors (prostaglandin E2 receptors 1–4, PAFR, LPAR1–6) and activate signaling pathways that promote cancer cell proliferation, survival, and migration.^[35]^

PEs are the second most abundant phospholipid in cells and play a crucial role in maintaining membrane integrity and fluidity.^[11,37]^ Alterations in PE levels may influence cancer cell membrane properties and signaling pathways involved in cell survival and proliferation.^[37,38]^ Furthermore, PE can be metabolized to generate bioactive lipids, such as lysophosphatidylethanolamine and N-acylethanolamines, which have been implicated in cancer progression.^[39–42]^ Lysophosphatidylethanolamine has been reported to be associated with invasion and metastasis in a variety of cancers,^[41,42]^ while N-acylethanolamines have been shown to exhibit anti-proliferative and pro-apoptotic effects in various cancer types.^[40,43]^

TGs are the main form of energy storage in the body and have been linked to obesity-related cancers.^[44]^ Elevated TG levels in cancer cells may provide an abundant energy source for their rapid proliferation and growth.^[45]^ The accumulation of TGs in cancer cells may also contribute to the formation of lipid droplets, which have been associated with increased cancer cell survival and resistance to chemotherapy.^[45]^ Additionally, TGs can be hydrolyzed to release free fatty acids, which can serve as signaling molecules and influence cancer cell metabolism, inflammation, and migration.^[46]^

SMs are sphingolipids crucial for cell adhesion, proliferation, migration, interaction, and death processes.^[47]^ Accumulation of specific SM species in cancer cell membranes can alter their biophysical properties, affecting lipid raft formation, protein trafficking, and signaling pathways in cancer progression.^[47]^ Moreover, SM can be metabolized to form bioactive sphingolipids like ceramide and sphingosine-1-phosphate (S1P), which have opposing effects on cancer cell survival.^[48]^ Ceramide, a pro-apoptotic lipid, induces cancer cell death by activating stress signaling pathways and mitochondrial dysfunction.^[48]^ Conversely, S1P, a pro-survival lipid, promotes cancer cell proliferation, migration, and angiogenesis through S1P receptor activation and downstream signaling pathways, such as PI3K/AKT and MAPK pathways.^[48]^

SEs are storage forms of cholesterol and have been implicated in cancer development due to their involvement in cholesterol homeostasis.^[49]^ Cholesterol is crucial for cell membrane structure and steroid hormone synthesis, which can stimulate the growth of hormone-dependent cancers like prostate cancer.^[49,50]^ Dysregulated SE levels in cancer cells may indicate altered cholesterol metabolism, leading to changes in membrane properties, antitumor immunity, and signaling pathways crucial for cancer progression.^[49,51]^ Moreover, the accumulation of cholesteryl esters in cancer cells is associated with increased lipid droplet formation and enhanced cancer cell survival.^[15]^ However, our findings, which show that SE (27:1/18:2) is linked to reduced prostate cancer risk, suggest that the role of SE in cancer may be complex and dependent on specific species.

PIs are a class of phospholipids that play critical roles in cell signaling, vesicle trafficking, and actin reorganization.^[52]^ These lipids can be phosphorylated at multiple positions on the inositol ring, generating various phosphoinositide species with distinct functions.^[53]^ Among the most well-studied phosphoinositides are phosphatidylinositol 4,5-bisphosphate (PI(4,5)P2) and phosphatidylinositol 3,4,5-trisphosphate (PI(3,4,5)P3), which regulate key signaling pathways such as the PI3K/AKT pathway.^[15,54]^ This pathway is frequently dysregulated in prostate cancer and plays a crucial role in cancer cell survival, proliferation, and migration.^[15]^ The tumor suppressor PTEN, which is often mutated or deleted in prostate cancer, acts as a negative regulator of the PI3K/AKT pathway by dephosphorylating PI(3,4,5)P3 back to PI(4,5)P2.^[15,54,55]^ Our finding that higher levels of PI(18:0_18:1) are associated with a reduced risk of prostate cancer suggests that this specific PI species may have a protective effect against prostate carcinogenesis. One possible mechanism is that PI(18:0_18:1) may compete with PI(4,5)P2 for PI3K, thereby reducing the generation of PI(3,4,5)P3 and attenuating the activation of the PI3K/AKT pathway. Alternatively, PI(18:0_18:1) may interact with other signaling molecules or effectors that negatively regulate prostate cancer cell survival and proliferation. Further research is needed to elucidate the precise molecular mechanisms through which PI(18:0_18:1) exerts its protective effect against prostate cancer.

In conclusion, various lipid species can influence tumor occurrence and development in the urinary system through diverse mechanisms, including modulating membrane properties, signaling pathways, energy metabolism, and cell death. Different molecular forms of the same lipid may play distinct roles. Further studies are needed to elucidate the specific molecular pathways through which these lipids affect cancer biology and to explore their potential as therapeutic targets for urologic cancers.

## 5. Strengths and limitations

The current study possesses several strengths. First, it utilized a large sample size, offering an unbiased perspective for causal assessment. Both the lipid and cancer samples were obtained from Finnish individuals, ensuring a homogeneous genetic background and minimizing potential population stratification bias. Additionally, comprehensive sensitivity analyses were performed, including heterogeneity tests, horizontal pleiotropy tests, and the leave-one-out method, enhancing the reliability and robustness of the results.

Nevertheless, this study also has several limitations that should be noted. Primarily, the study population was of European ancestry, which may limit the generalizability of the findings to other populations. Future research should include more diverse populations to validate the results across different ethnic and racial groups. Secondly, the study focused on a specific set of 179 lipid species, potentially overlooking other important lipid species. Expanding lipid coverage in future studies could provide a more comprehensive understanding of the plasma lipidome’s role in urinary system cancers. Lastly, there is a lack of basic research on the correlation between plasma levels of structurally diverse lipid species and urinary system cancers. Consequently, the specific biological mechanisms underlying the observed associations remain unclear. Further experimental studies are necessary to elucidate the molecular pathways through which these lipid species influence cancer development and progression.

## 6. Conclusion

In our study, the two-sample MR method was used to explore the relationship between the plasma lipidome and urological cancers. Several lipid species within the classes of PC, PE, TG, SM, and SE were identified that were associated with an increased or decreased risk of one or more urological cancers. Interestingly, it was also found that some of the same lipid species had consistent effects across different types of urologic cancers, suggesting that these lipids may play a broad role in urinary system cancers. Our findings provide novel insights into the potential role of the plasma lipidome in modulating cancer risk and underscore the importance of considering lipid structural diversity in cancer etiology. These results have significant implications for the early detection and prevention of urinary system cancers, as well as for the development of new therapeutic strategies targeting lipid metabolism. Further research is necessary to validate our findings in diverse populations, elucidate the underlying biological mechanisms, and explore the clinical utility of these lipid species as biomarkers or therapeutic targets in urinary tract cancers.

## Author contributions

**Conceptualization:** Rong Chen, Liujie He, Zhiyao Huang, Jie Sun, Qiang Wang.

**Data curation:** Rong Chen, Liujie He, Zhiyao Huang, Jie Sun, Qiang Wang.

**Formal analysis:** Rong Chen.

**Funding acquisition:** Qiang Wang.

**Methodology:** Rong Chen.

**Software:** Rong Chen.

**Supervision:** Qiang Wang.

**Visualization:** Rong Chen, Liujie He, Zhiyao Huang, Jie Sun.

**Writing – original draft:** Rong Chen.

**Writing – review & editing:** Rong Chen, Liujie He, Zhiyao Huang, Jie Sun, Qiang Wang.

## Supplementary Material


