# Supplementary material for: Causal relationship between the plasma lipidome and urological cancers: A two-sample Mendelian randomization study
Source: Medicine (Baltimore). 2025 Jun 6;104(23):e42577. doi: 10.1097/MD.0000000000042577 (PMC12151044; doi:10.1097/MD.0000000000042577)

Fig. S1: Forest plot displaying the effect of each significant lipid species on bladder cancer. An OR value greater than 1 is considered a risk factor for bladder cancer, while an OR value less than 1 is considered a protective factor.

lipidome	method	nsnp	pval	OR(95%CI)
Sterol ester (27:1/16:0) levels	MR Egger	34	0.0661	1.2540 (0.9932–1.5832)
	Weighted median	34	0.1337	1.1339 (0.9621–1.3364)
	Inverse variance weighted	34	0.0223	1.1483 (1.0199–1.2929)
	Simple mode	34	0.4076	1.1364 (0.8429–1.5323)
	Weighted mode	34	0.2326	1.1490 (0.9185–1.4373)
Phosphatidylcholine (18:0_20:3) levels	MR Egger	27	0.2268	1.2258 (0.8883–1.6917)
	Weighted median	27	0.0058	1.3004 (1.0791–1.5671)
	Inverse variance weighted	27	<0.001	1.2574 (1.1008–1.4363)
	Simple mode	27	0.0697	1.4290 (0.9872–2.0684)
	Weighted mode	27	0.0584	1.3598 (1.0031–1.8435)
Sphingomyelin (d38:1) levels	MR Egger	38	0.0617	1.2036 (0.9970–1.4529)
	Weighted median	38	0.1267	1.1217 (0.9680–1.2999)
	Inverse variance weighted	38	0.0225	1.1201 (1.0161–1.2347)
	Simple mode	38	0.6711	1.0602 (0.8113–1.3853)
	Weighted mode	38	0.2280	1.1254 (0.9317–1.3595)
Sphingomyelin (d40:2) levels	MR Egger	36	0.0298	1.3027 (1.0365–1.6372)
	Weighted median	36	0.1945	1.1233 (0.9423–1.3392)
	Inverse variance weighted	36	0.0118	1.1564 (1.0328–1.2948)
	Simple mode	36	0.8941	1.0213 (0.7509–1.3889)
	Weighted mode	36	0.4877	1.0859 (0.8625–1.3672)
Triacylglycerol (46:1) levels	MR Egger	22	0.0327	1.5107 (1.0621–2.1488)
	Weighted median	22	0.0686	1.2048 (0.9859–1.4722)
	Inverse variance weighted	22	0.0337	1.1775 (1.0127–1.3692)
	Simple mode	22	0.2958	1.2471 (0.8329–1.8674)
	Weighted mode	22	0.3100	1.2471 (0.8226–1.8906)
Triacylglycerol (50:5) levels	MR Egger	26	0.1869	1.1780 (0.9301–1.4920)
	Weighted median	26	0.0880	1.1670 (0.9773–1.3934)
	Inverse variance weighted	26	0.0106	1.1733 (1.0379–1.3265)
	Simple mode	26	0.2901	1.2060 (0.8587–1.6938)
	Weighted mode	26	0.2229	1.2160 (0.8949–1.6522)
Triacylglycerol (52:6) levels	MR Egger	24	0.1116	1.3184 (0.9508–1.8280)
	Weighted median	24	0.1540	1.1586 (0.9463–1.4185)
	Inverse variance weighted	24	0.0404	1.1611 (1.0065–1.3393)
	Simple mode	24	0.2285	1.2591 (0.8740–1.8139)
	Weighted mode	24	0.1829	1.2288 (0.9158–1.6488)

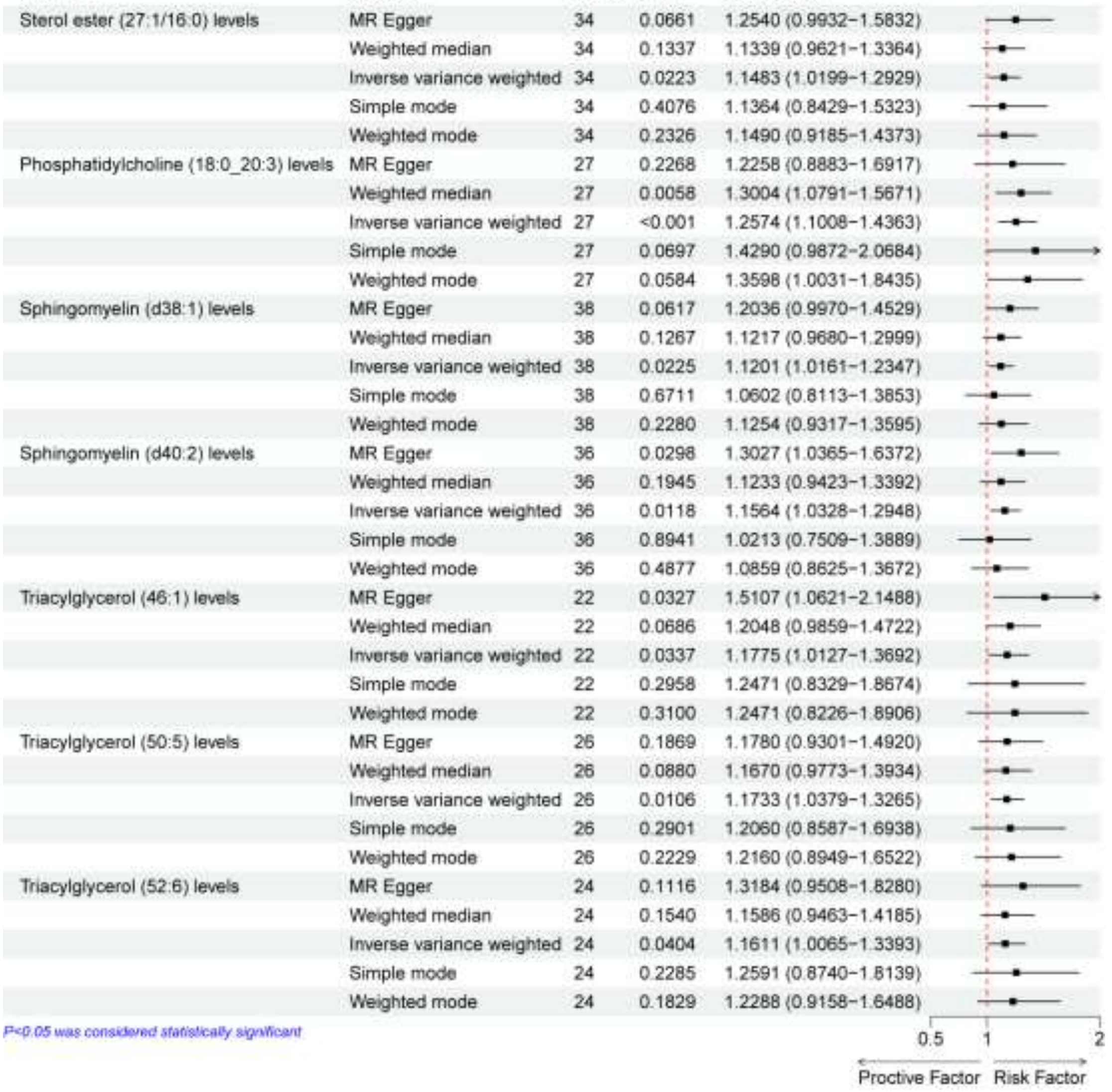

P<0.05 was considered statistically significant

Fig. S2: Scatter plot of the MR results for the causal relationship between liposomes and bladder cancer. (A). Sterol ester (27:1/16:0) levels, (B). Phosphatidylcholine (18:0_20:3) levels, (C). Sphingomyelin (d38:1) levels, (D). Sphingomyelin (d40:2) levels, (E). Triacylglycerol (46:1) levels, (F). Triacylglycerol (50:5) levels, (G). Triacylglycerol (52:6) levels.

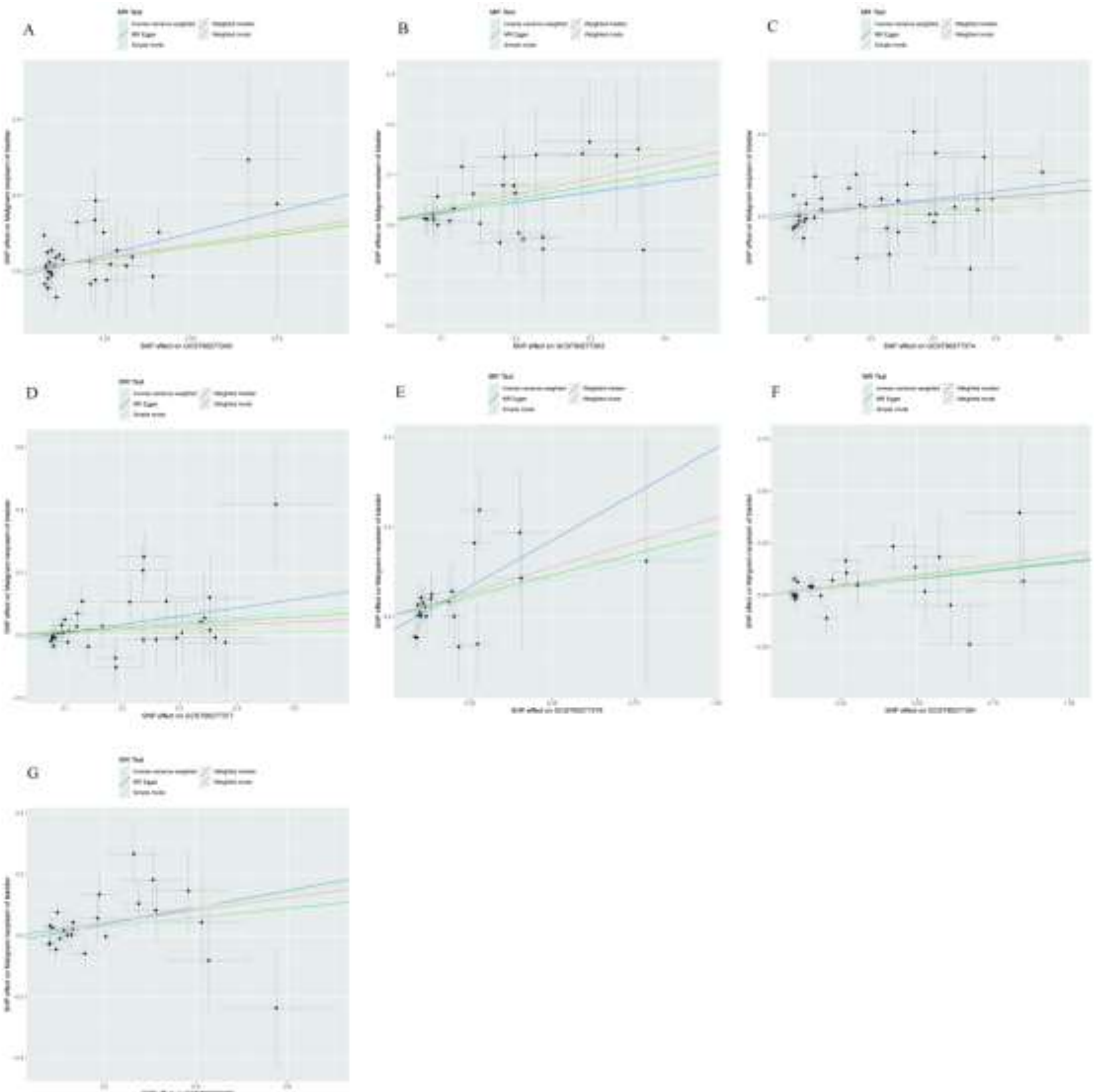

Fig. S3: Funnel plot of the MR results for the causal relationship between liposomes and bladder cancer. (A). Sterol ester (27:1/16:0) levels, (B). Phosphatidylcholine (18:0_20:3) levels, (C). Sphingomyelin (d38:1) levels, (D). Sphingomyelin (d40:2) levels, (E). Triacylglycerol (46:1) levels, (F). Triacylglycerol (50:5) levels, (G). Triacylglycerol (52:6) levels.

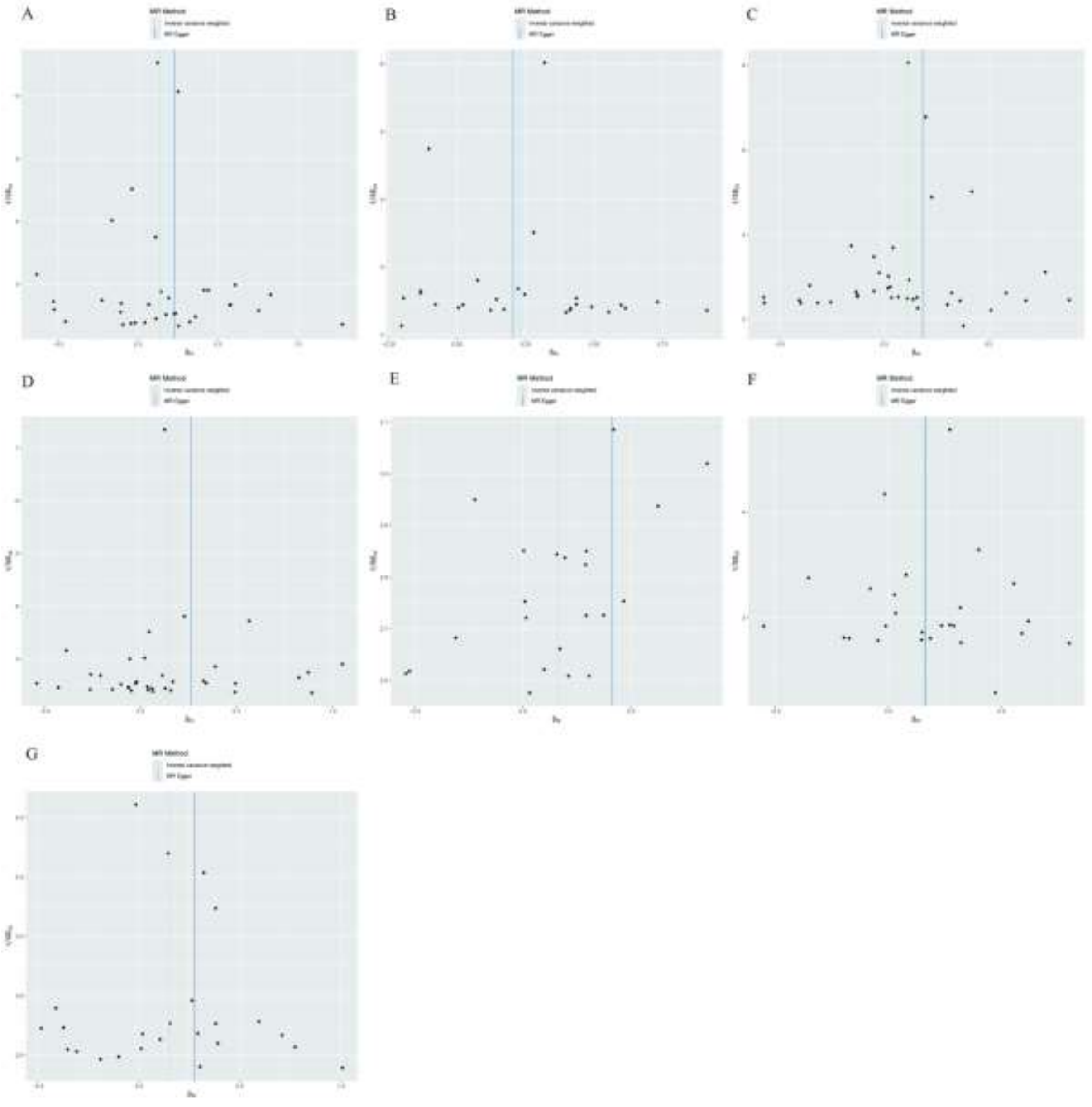

Fig. S4: Forest plot displaying the effect of each significant lipid species on kidney cancer. An OR value greater than 1 is considered a risk factor for kidney cancer, while an OR value less than 1 is considered a protective factor.

lipidome	method	nsnp	pval	OR(95%CI)
Phosphatidylcholine (16:0_22:6) levels	MR Egger	23	0.3579	1.1551 (0.8552−1.5601)
	Weighted median	23	0.0239	1.2468 (1.0296−1.5097)
	Inverse variance weighted	23	0.0311	1.1709 (1.0145−1.3516)
	Simple mode	23	0.1844	1.2943 (0.8950−1.8718)
	Weighted mode	23	0.1340	1.3190 (0.9306−1.8696)
Phosphatidylcholine (O−16:0_18:1) levels	MR Egger	22	0.4733	1.1534 (0.7866−1.6915)
	Weighted median	22	0.2148	1.1432 (0.9253−1.4125)
	Inverse variance weighted	22	0.0288	1.1775 (1.0171−1.3633)
	Simple mode	22	0.7944	1.0533 (0.7163−1.5488)
	Weighted mode	22	0.3948	1.1653 (0.8252−1.6454)
Phosphatidylcholine (O−16:0_20:4) levels	MR Egger	29	0.1720	1.1363 (0.9505−1.3582)
	Weighted median	29	0.0225	1.1831 (1.0240−1.3668)
	Inverse variance weighted	29	0.0372	1.1133 (1.0064−1.2315)
	Simple mode	29	0.1320	1.3119 (0.9310−1.8486)
	Weighted mode	29	0.0207	1.2111 (1.0392−1.4113)
Phosphatidylethanolamine (16:0_20:4) levels	MR Egger	25	0.6492	1.0390 (0.8829−1.2227)
	Weighted median	25	0.0940	1.1040 (0.9833−1.2396)
	Inverse variance weighted	25	0.0324	1.1013 (1.0081−1.2030)
	Simple mode	25	0.1196	1.1972 (0.9622−1.4896)
	Weighted mode	25	0.0847	1.1087 (0.9908−1.2406)
Phosphatidylethanolamine (18:0_20:4) levels	MR Egger	20	0.2427	1.1681 (0.9078−1.5030)
	Weighted median	20	0.0380	1.1600 (1.0083−1.3346)
	Inverse variance weighted	20	0.0014	1.2066 (1.0752−1.3541)
	Simple mode	20	0.0586	1.3684 (1.0081−1.8575)
	Weighted mode	20	0.2017	1.1052 (0.9529−1.2819)
Sphingomyelin (d32:1) levels	MR Egger	30	0.6932	1.0365 (0.8690−1.2362)
	Weighted median	30	0.0449	1.1700 (1.0036−1.3641)
	Inverse variance weighted	30	0.0372	1.1161 (1.0065−1.2375)
	Simple mode	30	0.0870	1.3197 (0.9709−1.7939)
	Weighted mode	30	0.0375	1.1845 (1.0172−1.3793)
Triacylglycerol (48:2) levels	MR Egger	24	0.3959	1.1621 (0.8271−1.6329)
	Weighted median	24	0.0318	1.2200 (1.0174−1.4629)
	Inverse variance weighted	24	0.0315	1.1682 (1.0139−1.3460)
	Simple mode	24	0.3494	1.1933 (0.8303−1.7151)
	Weighted mode	24	0.1533	1.3329 (0.9103−1.9516)

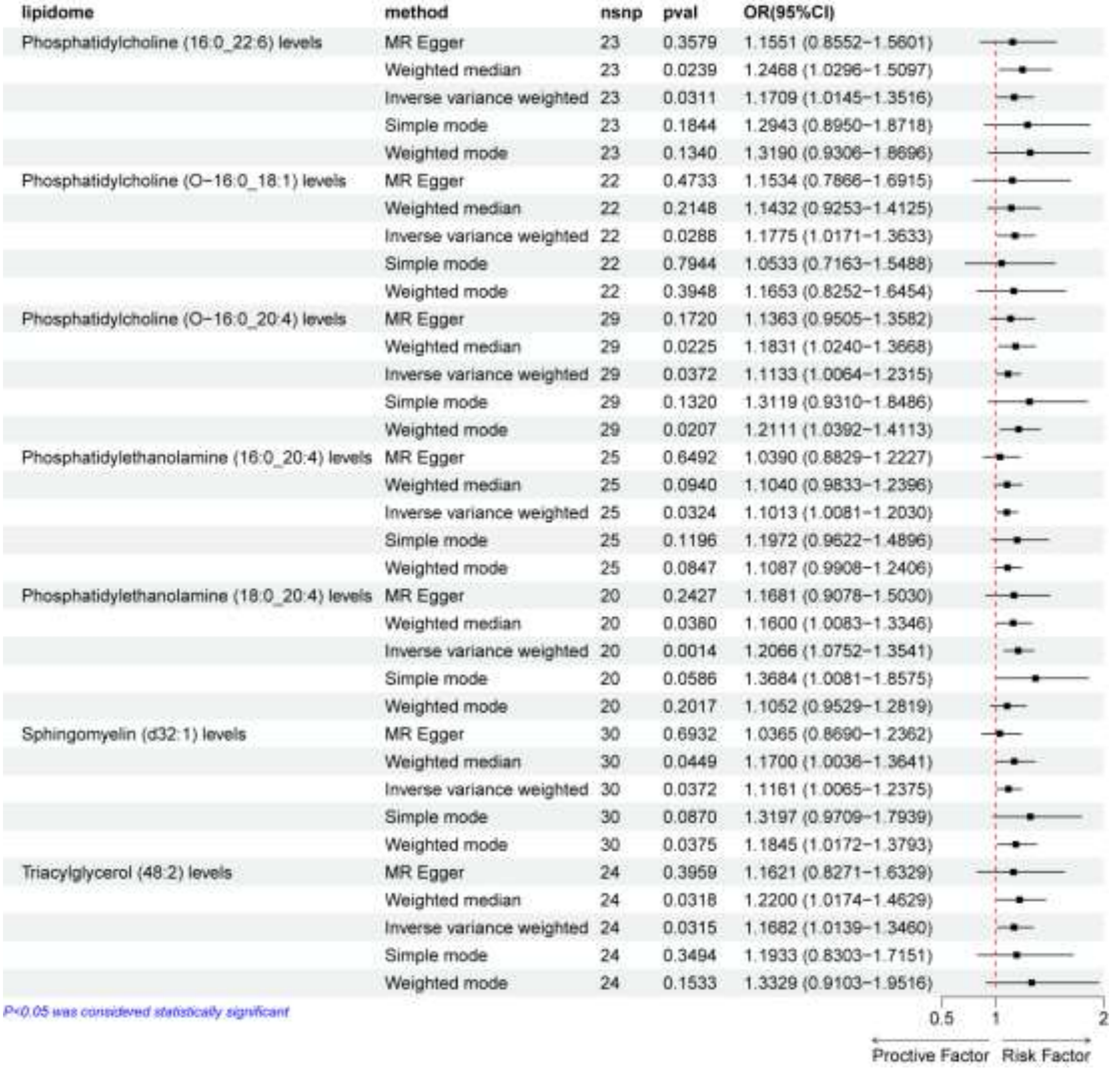

P<0.05 was considered statistically significant

Fig. S5: Scatter plot of the MR results for the causal relationship between liposomes and kidney cancer. (A). Phosphatidylcholine (16:0_22:6) levels, (B). Phosphatidylcholine(O-16:1_18:1) levels, (C). Phosphatidylcholine(O-16:0_20:4) levels, (D). Phosphatidylethanolamine (16:0_20:4), (E). Phosphatidylethanolamine (18:0_20:4) levels, (F). Sphingomyelin (d32:1) levels, (G). Triacylglycerol (48:2) levels.

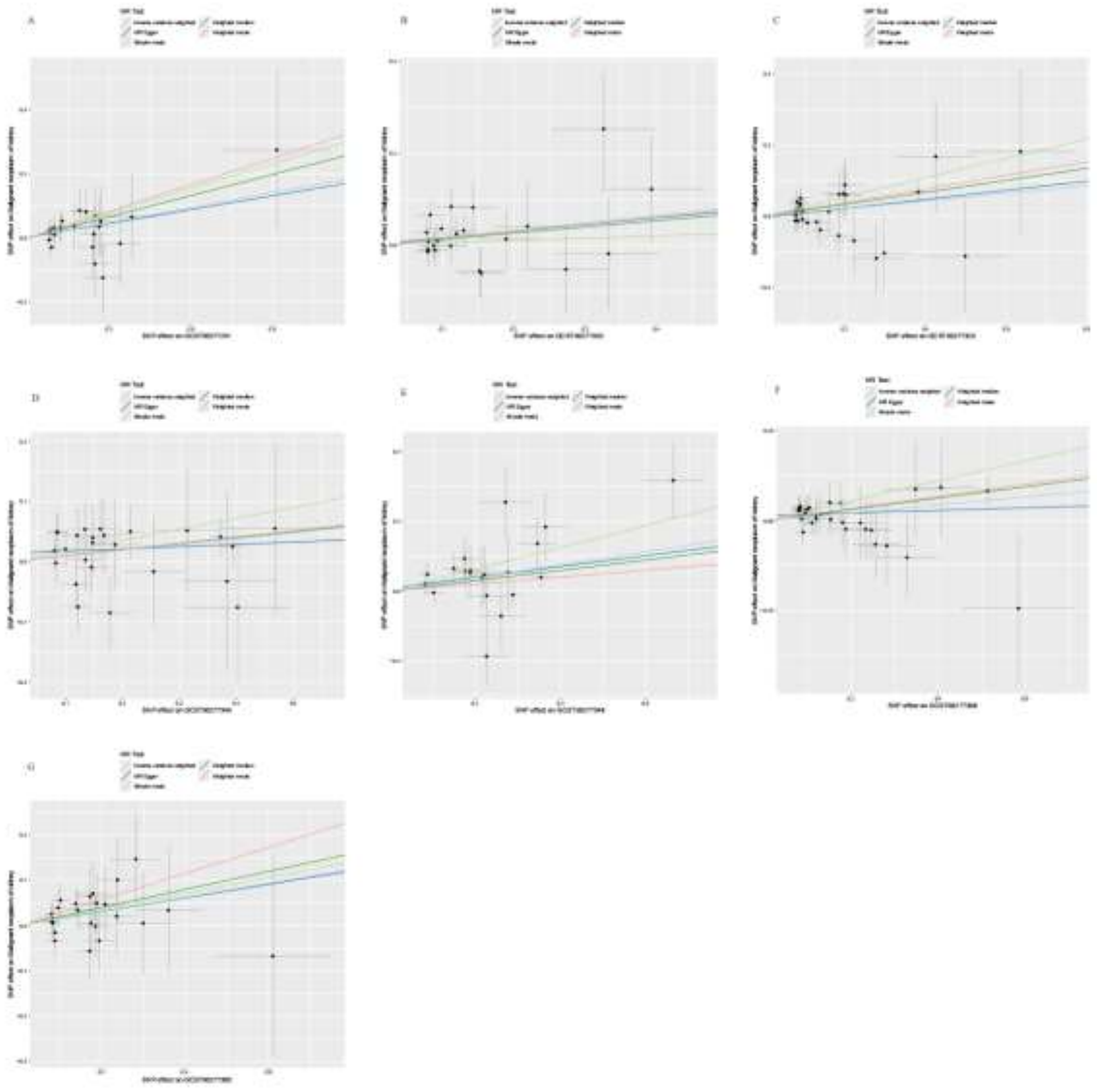

Fig. S6: Funnel plot of the MR results for the causal relationship between liposomes and kidney cancer. (A). Phosphatidylcholine (16:0_22:6) levels, (B). Phosphatidylcholine(O-16:1_18:1) levels, (C). Phosphatidylcholine(O-16:0_20:4) levels, (D). Phosphatidylethanolamine (16:0_20:4), (E). Phosphatidylethanolamine (18:0_20:4) levels, (F). Sphingomyelin (d32:1) levels, (G). Triacylglycerol (48:2) levels.

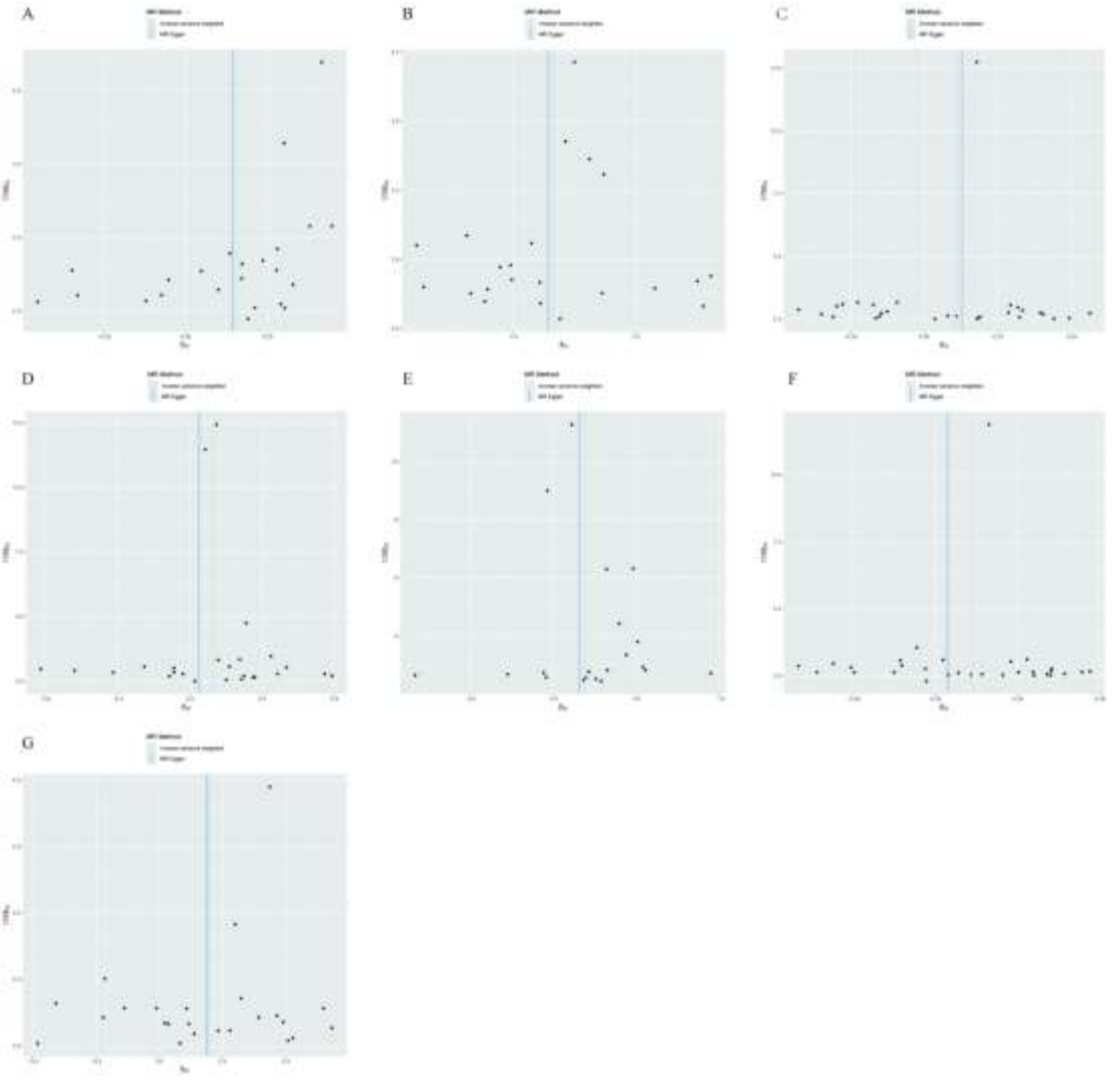

Fig. S7: Forest plot displaying the effect of each significant lipid species on prostate cancer. An OR value greater than 1 is considered a risk factor for prostate cancer, while an OR value less than 1 is considered a protective factor.

lipidome	method	nsnp	pval	OR(95%CI)
Sterol ester (27:1/17:0) levels	MR Egger	28	0.5471	1.0514 (0.8951−1.2349)
	Weighted median	28	0.0176	1.1096 (1.0183−1.2092)
	Inverse variance weighted	28	0.0477	1.0633 (1.0006−1.1299)
	Simple mode	28	0.1179	1.1629 (0.9683−1.3968)
	Weighted mode	28	0.1165	1.1459 (0.9720−1.3509)
Sterol ester (27:1/18:2) levels	MR Egger	24	0.1797	0.9332 (0.8463−1.0291)
	Weighted median	24	0.1718	0.9458 (0.8731−1.0245)
	Inverse variance weighted	24	0.0417	0.9453 (0.8955−0.9979)
	Simple mode	24	0.9844	0.9986 (0.8666−1.1506)
	Weighted mode	24	0.3914	0.9558 (0.8636−1.0578)
Sterol ester (27:1/20:3) levels	MR Egger	30	0.2452	1.0685 (0.9578−1.1920)
	Weighted median	30	0.1010	1.0665 (0.9875−1.1518)
	Inverse variance weighted	30	0.0474	1.0527 (1.0006−1.1075)
	Simple mode	30	0.7452	1.0249 (0.8847−1.1873)
	Weighted mode	30	0.3174	1.0620 (0.9458−1.1925)
Phosphatidylcholine (18:0_18:3) levels	MR Egger	23	0.8528	1.0142 (0.8757−1.1746)
	Weighted median	23	0.0565	1.0974 (0.9974−1.2074)
	Inverse variance weighted	23	0.0064	1.1133 (1.0306−1.2027)
	Simple mode	23	0.3023	1.1196 (0.9079−1.3806)
	Weighted mode	23	0.5120	1.0607 (0.8919−1.2616)
Phosphatidylcholine (18:0_20:3) levels	MR Egger	27	0.1513	1.1100 (0.9668−1.2744)
	Weighted median	27	0.0464	1.0859 (1.0013−1.1776)
	Inverse variance weighted	27	0.0031	1.0898 (1.0293−1.1538)
	Simple mode	27	0.1932	1.1001 (0.9564−1.2655)
	Weighted mode	27	0.0791	1.1100 (0.9925−1.2415)
Phosphatidylcholine (18:0_20:4) levels	MR Egger	27	0.1436	1.0388 (0.9887−1.0914)
	Weighted median	27	0.0302	1.0443 (1.0042−1.0860)
	Inverse variance weighted	27	0.0021	1.0550 (1.0196−1.0915)
	Simple mode	27	0.3259	1.0697 (0.9375−1.2206)
	Weighted mode	27	0.0417	1.0470 (1.0039−1.0920)
Phosphatidylcholine (O−16:2_18:0) levels	MR Egger	13	0.5672	1.0554 (0.8822−1.2626)
	Weighted median	13	0.4385	1.0401 (0.9415−1.1491)
	Inverse variance weighted	13	0.0466	1.0769 (1.0011−1.1584)
	Simple mode	13	0.6290	1.0432 (0.8826−1.2329)
	Weighted mode	13	0.2189	1.0880 (0.9578−1.2359)
Phosphatidylethanolamine (16:0_20:4) levels	MR Egger	25	0.2198	1.0488 (0.9740−1.1294)
	Weighted median	25	0.0173	1.0712 (1.0122−1.1335)
	Inverse variance weighted	25	0.0078	1.0560 (1.0144−1.0993)
	Simple mode	25	0.2110	1.0714 (0.9645−1.1901)
	Weighted mode	25	0.0575	1.0549 (1.0009−1.1118)
Phosphatidylethanolamine (18:0_20:4) levels	MR Egger	20	0.0241	1.1332 (1.0259−1.2517)
	Weighted median	20	0.0080	1.0820 (1.0208−1.1469)
	Inverse variance weighted	20	0.0061	1.0681 (1.0189−1.1196)
	Simple mode	20	0.1569	1.0768 (0.9759−1.1882)
	Weighted mode	20	0.0172	1.0836 (1.0202−1.1510)
Phosphatidylethanolamine (O−16:1_22:5) levels	MR Egger	17	0.0278	0.8444 (0.7370−0.9675)
	Weighted median	17	0.0450	0.9068 (0.8241−0.9978)
	Inverse variance weighted	17	0.0142	0.9140 (0.8506−0.9821)
	Simple mode	17	0.1989	0.8874 (0.7452−1.0568)
	Weighted mode	17	0.1902	0.8874 (0.7479−1.0530)
Phosphatidylinositol (18:0_18:1) levels	MR Egger	24	0.7767	0.9792 (0.8484−1.1302)
	Weighted median	24	0.1835	0.9490 (0.8785−1.0251)
	Inverse variance weighted	24	0.0238	0.9410 (0.8926−0.9920)
	Simple mode	24	0.0245	0.8411 (0.7305−0.9684)
	Weighted mode	24	0.3807	0.9550 (0.8633−1.0565)
Sphingomyelin (d40:2) levels	MR Egger	36	0.0829	1.1033 (0.9905−1.2291)
	Weighted median	36	0.4546	1.0313 (0.9512−1.1182)
	Inverse variance weighted	36	0.0324	1.0595 (1.0048−1.1171)
	Simple mode	36	0.8901	1.0103 (0.8742−1.1677)
	Weighted mode	36	0.6906	1.0206 (0.9237−1.1277)

P<0.05 was considered statistically significant

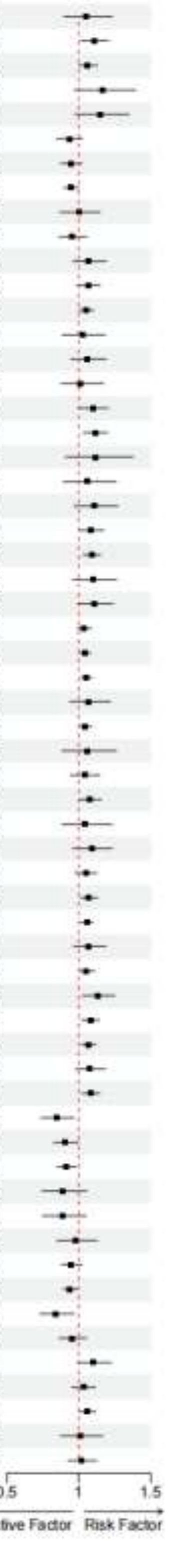

Fig. S8: Scatter plot of the MR results for the causal relationship between liposomes and prostate cancer. (A). Sterol ester (27:1/17:0) levels, (B). Sterol ester (27:1/18:2) levels, (C). Sterol ester (27:1/20:3) levels, (D). Phosphatidylcholine (18:0_18:3) levels, (E). Phosphatidylcholine (18:0_20:3) levels, (F). Phosphatidylcholine (18:0_20:4) levels, (G). Phosphatidylcholine(O-16:2_18:0) levels, (H). Phosphatidylethanolamine (16:0_20:4) levels, (I). Phosphatidylethanolamine (18:0_20:4), (J). Phosphatidylethanolamine(O-16:1_22:5) levels, (K). Phosphatidylinositol (18:0_18:1) levels, (L). levels Sphingomyelin (d40:2) levels.

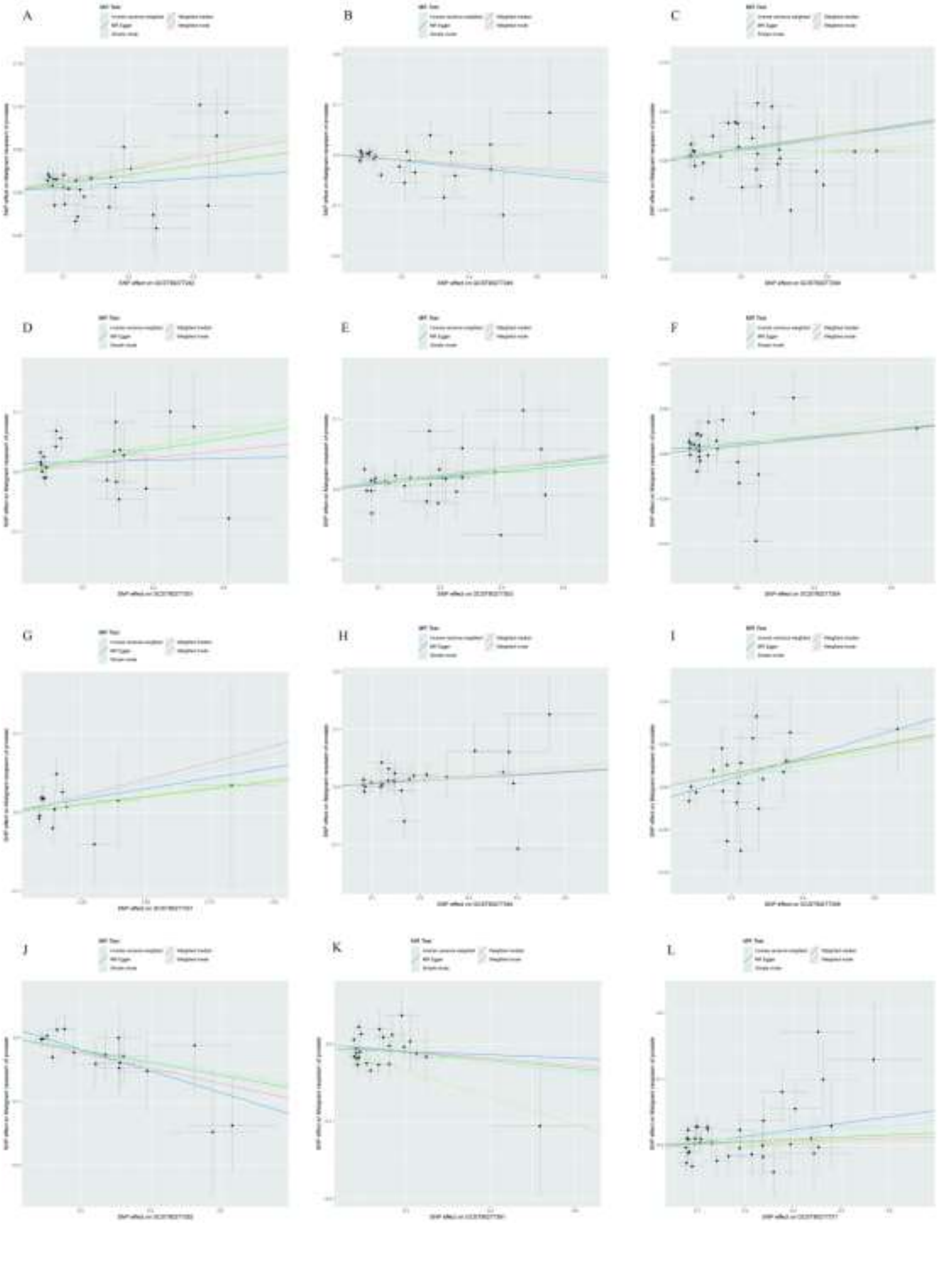

Fig. S9: Funnel plot of the MR results for the causal relationship between liposomes and prostate cancer. (A). Sterol ester (27:1/17:0) levels, (B). Sterol ester (27:1/18:2) levels, (C). Sterol ester (27:1/20:3) levels, (D). Phosphatidylcholine (18:0_18:3) levels, (E). Phosphatidylcholine (18:0_20:3) levels, (F). Phosphatidylcholine (18:0_20:4) levels, (G). Phosphatidylcholine(O-16:2_18:0) levels, (H). Phosphatidylethanolamine (16:0_20:4) levels, (I). Phosphatidylethanolamine (18:0_20:4), (J). Phosphatidylethanolamine(O-16:1_22:5) levels, (K). Phosphatidylinositol (18:0_18:1) levels, (L). levels Sphingomyelin (d40:2) levels.

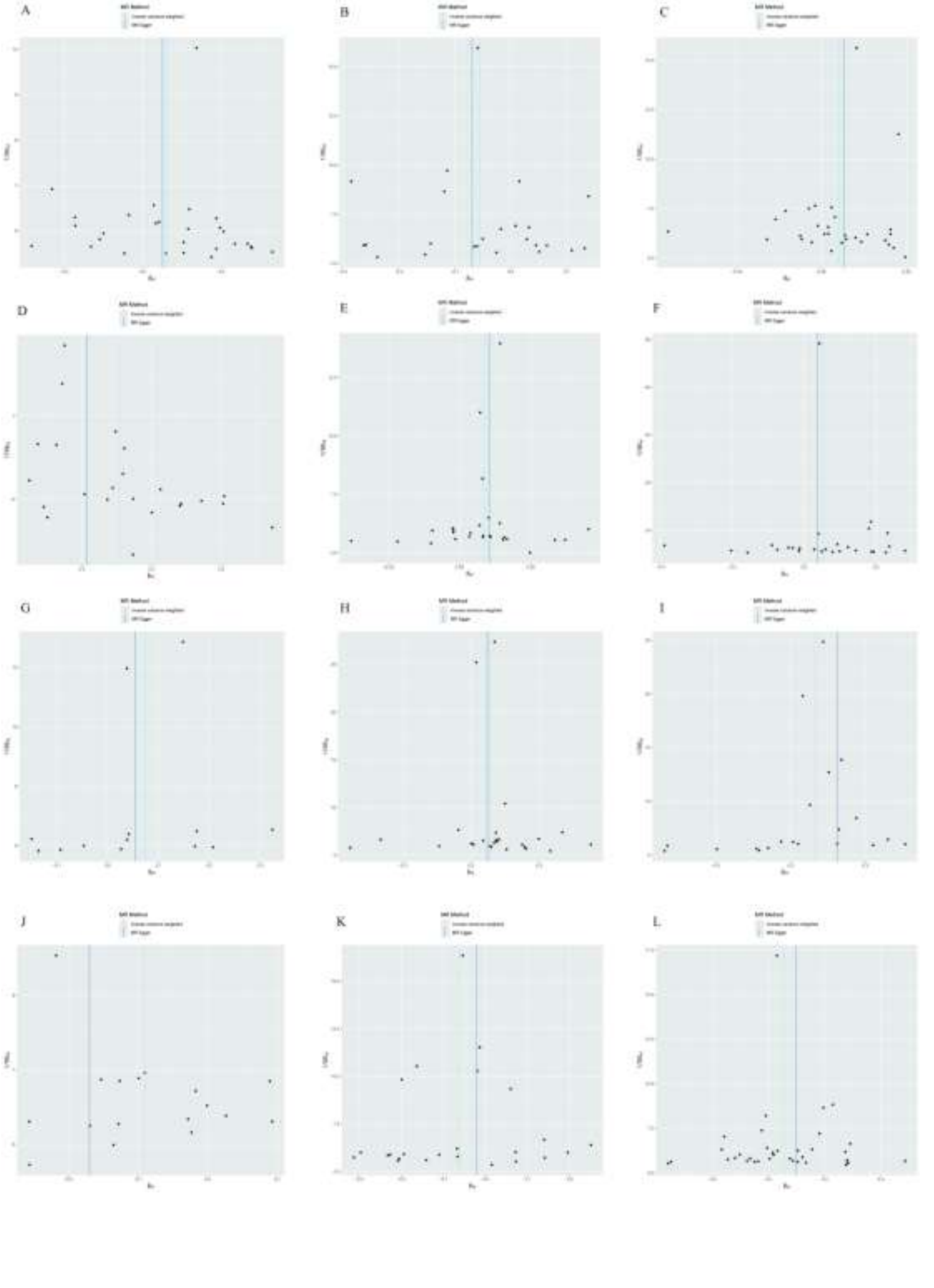

Supplement: Supplementary file 1 [file medi-104-e42577-s001.pdf]
